# Prevalence and Associated Risk Factors of Soil-Transmitted Helminth Infections among Schoolchildren around Lake Tana, Northwest Ethiopia

**DOI:** 10.1155/2022/4603638

**Published:** 2022-12-27

**Authors:** Melsew Getaneh, Tamirat Hailegebriel, Abaineh Munshea, Endalkachew Nibret

**Affiliations:** ^1^Gozamin Woreda Water and Energy Resource Development Office, East Gojam, Amhara Regional State, Ethiopia; ^2^Department of Biology, College of Science, Bahir Dar University, Bahir Dar, Ethiopia; ^3^Institute of Biotechnology, Bahir Dar University, Bahir Dar, Ethiopia

## Abstract

**Background:**

Soil-transmitted helminths (STHs) are among the most common neglected tropical diseases widely distributed in tropical countries with poor socioeconomic development. *Ascaris lumbricoides*, *Trichuris trichiura*, and hookworm are the three major STHs. This study aimed to determine the prevalence of STHs and associated risk factors among schoolchildren in selected primary schools around Lake Tana, Northwestern Ethiopia.

**Methods:**

A school-based cross-sectional study was conducted from February to May 2021 involving 337 study participants. A systematic random sampling method was utilized to select the study participants from the selected schools. Data related to sociodemographic characteristics of the study participants and risk factors for STH infections were collected using a pretested questionnaire. Stool samples were collected in sterile plastic containers from each participant and processed using Kato–Katz thick fecal smear and Ritchie's concentration techniques. The data were analyzed using the Statistical Package for Social Sciences software tool version 23, and factors with a *p* < 0.05 were considered as statistically significant.

**Results:**

The overall prevalence of STH infection was 38.3% (95% CI: 33.1–43.7). Hookworm infection was the predominant STH infection, which was detected in 26.1% schoolchildren followed by *A. lumbricoides* (14.8%) and *T. trichiura* (1.5%). Most of the study subjects (34.1%) had single infections, whereas only 4.2% study subjects had multiple helminthic infections. Among the risk factors considered in the study, lack of shoe wearing habit (adjusted odds ratio [AOR]=29.5; 95% confidence interval [CI]=6.59–132.55; *p* < 0.001), lack of knowledge on the prevention and control methods (AOR = 5.41; 95% CI = 2.44–11.98; *p* < 0.001), engagement in irrigation activities (AOR = 2.14; 95% CI = 1.02–4.57, *p* = 0.049), lack of toilet (AOR = 3.06; 95% CI = 1.31–7.16; *p* = 0.01), children grades of 5–8 (AOR = 2.62; 95% CI = 1.26–5.43; *p* = 0.01), playing on soils (AOR = 5.90; 95% CI = 2.79–12.49; *p* < 0.001), lack of fingernail trimming habit (AOR = 3.21; 95% CI = 1.57–6.55; *p* = 0.001), and male gender (AOR = 2.28; 95% CI: 1.19–4.39; *p* = 0.013) were significant explanatory factors for STH infection among schoolchildren in the study area.

**Conclusions:**

The present study showed that STHs were common among schoolchildren around Lake Tana. Therefore, education on personal and environmental hygiene should be taken into account to reduce the prevalence of STH infection in the study area.

## 1. Introduction

Soil-transmitted helminthic (STH) infections are among the most prevalent and widespread chronic human infections worldwide [1]. The most common STHs are *Ascaris lumbricoides*, *Trichuris trichiura*, and hookworms (*Necator americanus* and *Ancylostoma duodenale*). According to World Health Organization (WHO) 2022 report, about 1.5 billion people are infected with STH worldwide [2]. Pullan and Brooker estimated that 5.3 billion people, including 1.0 billion school-aged children, lived in areas of stable transmission for at least one STH species in 2010 [3]. STH infections are the greatest public health burden occurring in the developing world, namely sub-Saharan Africa [4, 5], South East Asia [1, 6], Latin America, and the Caribbean [7, 8]. The majority of these infections result from a low standard of living, poor socioeconomic status, and poor personal and environmental sanitation [1, 9].

STH infection is often associated with behavioral, environmental, and socioeconomic factors [10, 11]. STHs also cause impaired childhood growth and cognitive development and worsen school performance [12]. STH infection is suggested to be not only associated with poor school performance but also with high illness and death of schoolchildren [10]. More than half of all deaths from communicable diseases occur globally in school-age children (SAC) from 5 to 15 years of age [12]. These age groups are also more vulnerable to STH infection. The burden and severity of these diseases are higher in children due to their elevated susceptibility to potential risk factors of STH infections and their weak body resistance to parasitic load than in adults [13].

STH infection is a worldwide public health concern and the major cause of human disease, particularly in sub-Saharan Africa including Ethiopia. The Ethiopian government launched mass drug administration (MDA) for the control of STH and schistosomiasis in 2015 [14] and water, sanitation, and hygiene (WASH) programs. Despite these prevention and control programs, the prevalence of STH is still moderate to high in different localities of Ethiopia [15–19]. The prevalence and intensity of STH infections vary extremely from place to place as well as among various communities. SAC who live in warm and moist environments are more vulnerable to STH infection [20]. Warm and moist environmental conditions around water bodies, such as lakes and rivers, favour the long-term existence of eggs and larval stages.

Amhara region is one of the endemic areas for STH in Ethiopia. Studies in different parts of the Amhara region indicate moderate to high prevalence of STH among schoolchildren [15, 21]. Lake Tana is located at the center of Amhara region and the surrounding areas of the lake are suitable for STH and schistosomiasis transmission. Despite these, the numbers of studies targeting STH among schoolchildren are very limited and targets few schools [22–24]. In addition, moderate to high prevalence of STH was reported from fishermen [25] and Ethiopia Orthodox church students [21] around Lake Tana, Amhara Regional State, Ethiopia. Epidemiological studies on the prevalence of STH among high-risk groups are vital for policy makers to design appropriate prevention and control programs. Therefore, the present study aimed to determine the prevalence and associated risk factors of STH infections among schoolchildren in selected primary schools around Lake Tana.

## 2. Material and Methods

### 2.1. Description of Study Area

**Figure 1:**
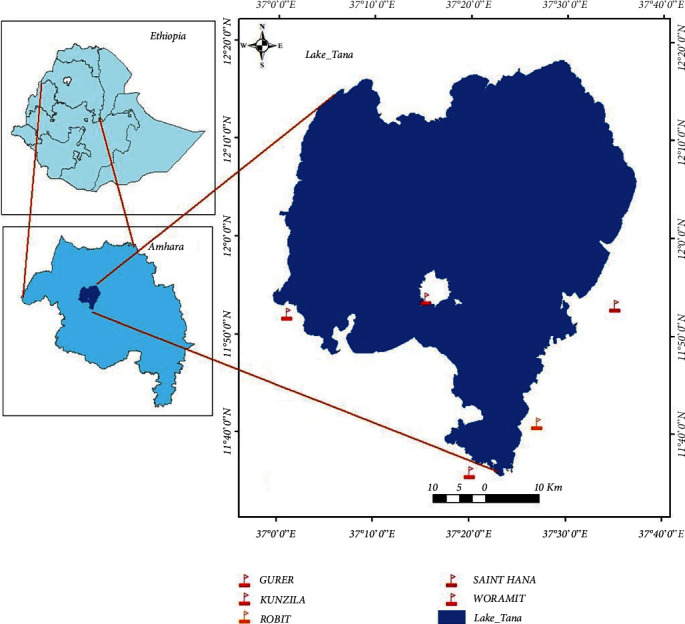
Map of the study area showing all selected primary schools around Lake Tana. The map was prepared using the ArcGIS online software.

The study was conducted in five selected full-cycle (Grades 1–8) primary schools in and around Lake Tana, Northwestern Ethiopia, namely Robit, Kunzila, Woramit, Gurer, and St. Hana primary schools (Figure 1). These schools are located between 11°36′latitude N and 37°35′longitude E. All the selected schools are found around Lake Tana. Lake Tana is the largest lake by surface area of (3,156 km^2^) in Ethiopia, covering about 50% of total fresh water in Ethiopia, and it is located about 570 km away from Addis Ababa. Lake Tana is a shallow lake with a mean depth of 8 m and a maximum depth of 14 m. Seven large permanent rivers and about 40 small seasonal rivers feed the lake. The lake has a warm temperature climate and a mean annual temperature of 13.5°C–27.7°C, and the mean annual rainfall is about 1,500 mm. The average annual temperature is 20.4°C. The peak amount of rainfall occurs in June and July.

### 2.2. Study Design

A school-based cross-sectional study was conducted from February to April 2021 to assess the prevalence of STH infections and their associated risk factors among students who attended the selected primary schools around Lake Tana, Northwest Ethiopia.

### 2.3. Source and Study Population

All students attending the five full-cycle primary schools (Robit, Kunzila, Woramit, St. Hana, and Gurer) were considered as the source population. The total number of students attending during the 2020/2021 academic year in these schools was 4,660 (Male = 2,250 and Female = 2,410). Students attending classes in the five selected schools and volunteers to participate in the study were considered as the study population.

### 2.4. Sample Size Determination and Sampling Technique

#### 2.4.1. Sample Size Determination

The sample size of this study was determined using the single proportion formula as described by Naing et al. [26] by considering 27.5% prevalence (*p*) of STH from the previous study in the area [23], 5% marginal error (*d*), and 95% confidence level (*Z* = 1.96).

(1) $$\begin{array}{c} n=\frac{{\left(1.96\right)}^{2}\times 0.275\;\left(1-0.275\right)}{{\left(0.05\right)}^{2}}=306\;n=\frac{Z^{2}P\;\left(1-P\right)}{d^{2}}. \end{array}$$

To minimize errors arising due to the non-response rate, 10% was added to the calculated sample size. As a result, a total of 337 schoolchildren were chosen to participate in the study.

#### 2.4.2. Sampling Technique

A systematic random sampling method was used to select 337 study participants. By using the sample proportion formula, a quota was allocated for all selected primary schools. The sample size (*n*) was proportionally divided by the total number of children in each school. The actual number of students selected from each school was obtained by multiplying the total number of students in each school multiplied by the selection factor. Hence, the number of students selected from Robit, Woramit, Gurer, Dek, and Qunzela was 105, 70, 20, 67, and 75, respectively.

The total number of students in the selected five schools was divided by the sample size obtained to determine the interval for selection (4,660/337 = 14) of study participants. The lottery method was used to select the first participant, and selection was continued at every 14th interval throughout the whole sections of all schools. The study participants were selected from each grade and section using systematic random sampling by considering the class attendance list or roster as a sampling frame. The 337 students were selected until the last interval of the last section.

### 2.5. Inclusion and Exclusion Criteria

Voluntary schoolchildren and their guardians/caretakers who were willing to provide written consent for the inclusion of their children in this study were included in the study. Besides, schoolchildren who did not receive anti-helminthic treatment during the study period were included. Schoolchildren that were not voluntary for the provision of stool samples for examination or those who had an anti-helminthic drug for two weeks were excluded from the study.

### 2.6. Study Variables

The dependent variable in this study was the infection status of STHs infection. The independent variables include gender, age, grade level, residence, educational level of parent, source of drinking water, latrine system, family size, hand washing habits, playing on soil, eating raw-vegetable, shoes wearing habits, knowledge on the transmission of STH, and personal and environmental hygiene.

### 2.7. Sample Collection and Processing

A structured questionnaire was prepared in English and translated into the local language (Amharic) before the interview. A full explanation of the objectives of the study was given to all voluntary participants, and they were counselled about STHs by the laboratory technologist. After obtaining written informed consent from students and their parents/guardians; sociodemographic, behavioral, and environmental risk factors of STH infections were collected from each selected student. All selected students were provided with a labelled stool cup with a clean wooden applicator stick to bring about 2 g of fresh stool sample of their own. All collected stool samples were processed using Kato–Katz thick smear and Ritchie concentration at the school compound immediately after collection by trained laboratory technologists.

The Kato–Katz thick smear was employed by taking 41.7 mg fecal material from an individual student as described elsewhere [27]. Eggs counts were not performed immediately at the spot due to lack of electricity in some of the schools. All eggs of STHs were counted from each template and converted to an egg per gram (EPG) of feces by multiplying with 24. The intensity of STH infections was classified as light, moderate, and heavy infections based on WHO guidelines [28], as indicated in Table 1. The remaining stool sample from Kato–Katz's thick fecal smear was processed using Ritchie's concentration technique [29]. The detailed procedure of Ritchie's concentration used in this study was described elsewhere [30].

### 2.8. Quality Control

To ensure reliable information, laboratory chemicals and consumables were properly checked, and quality control was performed before data collection. The study questionnaire was pre-tested in 5% of schoolchildren from other schools in the nearby areas but not included in the study. About 10% of the processed samples were re-examined by experienced laboratory technologists who did not have prior knowledge about the result. The result of the examination served as quality control.

### 2.9. Data Analysis

All collected data were analyzed using Statistical Package for Social Sciences (SPSS) statistical software tool version 23. Descriptive statistics were used to calculate the frequency and percentage of sociodemographic characteristics of the study participants. The association between STH infection and sex, grade level, family size, residence, source of drinking water, fingernail cleaning habit, shoe-wearing habit, and the effects of personal and environmental hygiene were analyzed by using univariate logistic regression analysis. Risk factors with a *p* < 0.25 in the univariate logistic regression analysis were selected and entered into a multivariable logistic regression model [31]. Multivariate logistic regression analysis was used primarily to identify the major explanatory factor(s) among the sociodemographic and behavioral factors considered in the present study with STH parasitic infections. *p-*Values of less than 0.05 were considered statistically significant.

### 2.10. Ethical Consideration and Consent to Participate

The study was conducted after obtaining ethical clearance from the Ethical Review Committee of Science College, Bahr Dar University, with Ref No: Post Graduate and Research Vice-Dean (PGRCSVD)/155/2020. After getting ethical clearance, the objective of the study was informed to the school community, and written informed consent was obtained from parents/guardians of all selected students. Participation in the study was voluntary basis, and study subjects were free to withdraw from the study at any time without losing the benefits they are supposed to obtain from the school. All schoolchildren who were found positive for intestinal parasites were treated by health officers found in the nearby health centers free of charge.

## 3. Results

### 3.1. Sociodemographic Characteristics

A total of 337 students were selected and invited to collect sociodemographic information and stool samples. All the selected students (female = 178 and male = 159) provided complete information and appropriate stool samples. The majority of the students were Orthodox Christians (97.9%), living in rural areas (81%), borne from illiterate families (71.8%), and farmer families (68.8%). Similarly, most of the students belonged to the age group of 10–14 years (66.2%) and were in grades 1–4 (59.6%; Table 2). The average age of the students was 11.3 ± 2.65.

### 3.2. Prevalence of STH among Schoolchildren

Out of the total 337 study participants, 129 (38.3%) were infected with one or more of the three different types of STHs (Table 2). The parasitic helminths identified in the study include hookworm with a prevalence of 26.1% (88/337), *A. lumbricoides* 14.2% (48/337), *T. trichiura* 1.5% (5/337), *Hymenolepis nana* 1.2% (4/337), and *Enterobius vermicularis* 0.6% (2/337). Regarding the prevalence of STH among schools, the highest prevalence of STH was observed among schoolchildren in Robit Primary School, whereas the lowest prevalence was observed in Gurer Primary School (Table 3). The prevalence of STH was higher among male gender (55.8%) than the female gender (44.2%). One hundred seventeen students (34.7%) of the study subjects had a single helminthic infection, whereas twelve students (3.6%) had double infections of hookworm with *A. lumbricoides* or hookworm with *T. trichiura.*

**Figure 2:**
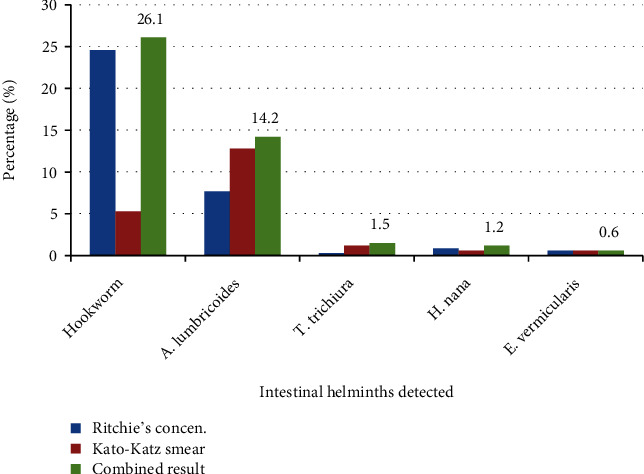
Prevalence of soil-transmitted helminth and other intestinal parasites detected using Kato–Katz and Ritchie's concentration techniques among primary schoolchildren around Lake Tana, 2021.

The present study revealed that five helminths were detected among schoolchildren in the selected primary schools (Figure 2). Among these parasites, hookworm (26.1%) was the most dominant helminth followed by *A. lumbricoides* (14.2%) and *T. trichiura* (1.5%).

### 3.3. Infection Intensity of STH among Schoolchildren

Among 141 students positive for STHs, 65 students (*A. lumbricoides* = 43, hookworm = 18, and *T. trichiura* = 4) were positive for STH using Kato–Katz thick fecal smear. The arithmetic mean of EPG of fecal sample for *A. lumbricoides*, hookworm, and *T. trichiura* was 607.26, 177.33, and 72.00, respectively. All hookworm, *T. trichiura*, and 39 (90.7%) of *A. lumbricoides* were categorized as light infection intensity as per WHO classification [32]. The remaining 4 (9.3%) of schoolchildren had *A. lumbricoides* infection was classified as moderate infection (Table 1).

### 3.4. Factors Associated with STH Infection among Schoolchildren

A logistic regression model was used to identify potential explanatory variables associated with STHs infection. Univariate logistic regression analysis showed that gender of students, grade level, residence, open space defecation, eating of unwashed vegetables, participation in irrigation activities, shoes wearing habit, availability of toilet, washing hands after toilet, playing on contaminated soil, lack of knowledge about the route of transmission, and prevention and control methods of STHs were significantly associated with STHs infections in the study area using univariate analysis (Table 4).

Variables with a *p* < 0.25 in the univariate logistic regression model were selected and included in the multivariable logistic regression model. Multivariate logistic regression analysis revealed that sex of students, grade level, shoes wearing habits, lack of toilet availability, washing hands after toilet and before the diet, playing on contaminated soils, lack of knowledge about the prevention and control methods, and finger trimming habits were independent explanatory risk factors for STHs infections among schoolchildren in the study areas (Table 4).

The odds of male students infected with STHs were 2.3-fold higher than their female counterparts (adjusted odds ratio [AOR] = 2.29; 95% confidence interval [CI]: 1.19–4.39, *p* = 0.013). Similarly, students from 5 to 8 grades were 2.6 times at higher risk of infection than students of lower grades (AOR = 2.62; 95% CI: 1.26–5.43, *p* = 0.01). The odds of infection with STHs were about 5.4 times higher (AOR = 5.41, 95% CI: 2.44–11.98, *p* < 0.001) among students who had poor knowledge of the prevention and control methods of STHs infections.

Schoolchildren who had no shoes wearing habits were about 30-fold (AOR = 29.57, 95% CI: 6.59–132.55, *p* < 0.001) more likely infected with STHs than those schoolchildren who always wore shoes. Schoolchildren who lacked toilets in their backyards were about 3 times (AOR = 3.06; 95% CI: 1.31–7.16, *p* = 0.01) at higher risk of STHs infections compared with students who had toilets. Students who did not have a fingernail trimming habit were about 3 times more likely infected with STH than students who always trimmed their fingernails (AOR = 3.21, 95% CI: 1.57–6.55, *p* = 0.001). The odds of occurrence of STHs infection in schoolchildren who played with soil were about 6 times higher than those schoolchildren who did not play with soil (AOR = 5.90, 95% CI: 2.79–12.49, *p* < 0.001). Likewise, schoolchildren who participated in irrigational activity were about twofold (AOR = 2.14, 95% CI: 1.002–4.57, *p* = 0.049) more likely infected with STHs compared with students who did not participate in irrigation activity.

## 4. Discussion

Soil-transmitted helminthiasis is a serious public health concern in several tropical and sub-tropical countries with depleted resources, poor socioeconomic status, and personal hygiene. The burden of STH is high in SAC and is associated with high morbidity, mortality, and economic loss to endemic countries [20, 33]. Thus, knowledge about the distribution and prevalence of STH in a given community is important to identify vulnerable groups and design appropriate intervention programs. Thus, this study attempted to assess the prevalence of STH infections and their associated risk factors among schoolchildren in selected primary schools around Lake Tana, northwest Ethiopia.

The Ethiopian government launched MDA-based intervention to control and prevent STH and schistosomiasis from school-aged children in 2015. However, this study revealed that about 38% of schoolchildren in areas under MDA program were still positive for one or more STH infections. Similar to our finding, moderate to high prevalence of STH was reported in Ethiopia [34–37], Myanmar [38, 39], Kenya [40], India [41], and Nigeria [42]. In contrast, a lower prevalence of STH was reported in several studies in Ethiopia [43–46], Thailand [47], and Kenya [48]. These differences might be due to the differences in parasitological methods used, sample size, drinking water sources, level of personal hygiene, family size, family's educational status, and environmental conditions. In addition, although there is an ongoing MDA program, there might be frequent reinfection as a result of poor environmental sanitation related to open space defecation. The toilet coverage in Ethiopia is one of the lowest in sub-Saharan Africa. About 52% Ethiopian population used unimproved latrine service and most of them practice open space deification [49].

In the present study, we used two diagnostic methods Kato–Katz and Ritchie's concentration approaches to improve the chance of detection of STH infection. Kato–Katz thick smear should be examined and ready within sixty minutes of smear preparation, especially for hookworms [50]. However, most of the schools lack electricity and are unable to read Kato–Katz smear within this time limit, which might underestimate hookworm infection. To compensate this we used Ritchie's concentration to increase the detection of hookworm infections. In line with this higher prevalence of hookworm infection was observed using Ritchie's concentration technique than Kato–Katz fecal smear. A combination of methods generally gives a better detection rate and is recommended for the detection of parasitic infections [35, 51, 52].

As a general trend, *A. lumbricoides* is the most prevalent intestinal helminth in tropical and sub-tropical countries [53–55]. However, the present study revealed that hookworm was the predominant intestinal helminth followed by *A. lumbricoides*, whereas *T. trichiura* was the least prevalent STH in the study area. Similar observations were reported from studies conducted in Lao People's Democratic Republic [56], Nigeria [57], and Ethiopia specifically in the Amhara region [35, 58], in which this study was conducted. This could be the fact the high degree of soil contamination with night soil, high duration of soil contact, and shoes wearing habits of the study subjects. This study was conducted around Lake Tana, which is suitable for the long-term survival of hookworm larvae in the soil. In addition, most of the study subjects in the study area had a habit of walking barefooted. Therefore, the high prevalence of hookworm infection in the study area might be associated with the above-mentioned facts.

The prevalence of hookworm infection in this study was 26.1%, which is in agreement with studies reported in Amhara Region, Ethiopia [59, 60], India [41], Loa Peoples Democratic Republic [56], and Kenya [61]. In contrast, a high prevalence of hookworm infections was reported from various parts of Ethiopia [24, 62, 63], Ghana [64], and Nigeria [42]. This variation might be associated with differences in environmental conditions and socioeconomic and behavioral factors of the residents.

*A. lumbricoides* was the second most prevalent STH identified in this study, which is in line with studies reported from Ethiopia [62, 63], Myanmar [39], Nigeria [65], and Malaysia [66]. On the other hand, a high prevalence of *A. lumbricoides* was reported in school-based studies from different parts of Ethiopia [34, 36, 67], India [41], Nigeria [42], and Sri Lanka [68]. Such differences might have arisen due to differences in the environmental condition, personal hygiene, and sanitation of the study subjects across study sites. Poor sanitary conditions, the lifestyle of study participants, and socioeconomic conditions might be linked with high STH infection in Ethiopia, and elsewhere in sub-Saharan Africa.

Logistic regression analysis was employed to assess the degree of association of sociodemographic, environmental, and other potential risk factors of STH infection. The analysis revealed that gender and grade level of students, knowledge of prevention and control methods, shoes wearing habits, availability of toilets, playing on soil, and fingernail trimming habits were important explanatory variables of STH in the study area.

Students who did not wear shoes at all were nearly thirty times more likely infected with STH than students who frequently wear protective shoes. Similar associations between STH and shoes wearing habits were reported in Ethiopia [46, 69, 70], Kenya [48], and Nicaragua [71]. Shoe-wearing habits are the major risk factors for hookworm infection. Hookworm filariform larvae directly penetrate the intact skin of children when they are walking or playing on contaminated soils on barefoot. This study suggested that awareness-raising about personal hygiene in particular shoe-wearing habits may significantly change the level of hookworm infection in schoolchildren in Ethiopia.

Students who lacked basic knowledge of the prevention and control methods of STHs were about five times more likely infected with STHs than those students having basic knowledge of the prevention and control methods. Basic knowledge of the prevention and control methods of STH is vital to reducing the transmission of helminths as reported in several studies [72–74]. Most STHs are transmitted via contaminated soil. Therefore, basic information on the control and prevention methods of STH is important for preventive strategies.

The odds of infection by STHs in schoolchildren who were playing on soils were nearly six times higher risk of STHs infections than those who did not have this habit. A similar association between playing on soils and STHs infection was reported in Indonesia [75] and Ethiopia [22]. Likewise, children who did not trim their fingernails were about threefold at higher risk of STHs infections than those students who regularly trimmed their fingernails. The association between fingernail trimming habits and intestinal helminths infection has been well documented in Ethiopia [22, 46, 69], Indonesia [76], and Nigeria [77]. It is a well-known fact that the risk of STH infection is quite high among students who do not trim their fingernails and among those who play with contaminated soil due to the deposition of eggs in their fingernails.

The odds of STHs infections among children who lack toilets in their backyards were about threefold higher than among those students who had a toilet in their backyards. A similar observation was reported in Ethiopia [34, 46] and Nigeria [42]. Schoolchildren who lacked toilets in their backyard were forced to defecate in open fields, which might lead to a higher probability of contact with contaminated soil. These situations play vital roles in the transmission of STHs.

The other factor that was found to increase the likelihood of STH infection among children was participation in irrigation. Children who engaged in irrigational activities were about two times more likely to acquire STHs infections when compared with those children who did not involve in any irrigational activities. The association of STH infection and involvement in irrigational activities is reported from Ghana [78]. The high prevalence of STH infection among students who participated in irrigation might be associated with a high chance of contact with contaminated soils and the acquisition of eggs in their fingernails as fecal materials might be used as fertilizers in some of the farms.

The present study showed that the sex of students is one of the explanatory risk factors for the observed prevalence of STHs infections. Male students were a twofold higher chance of being infected than their female counterparts. Similar associations of gender with STH are reported from Nicaragua and Thailand [79, 80]. The high prevalence of STH infection among male students might be associated with their engagement in outdoor activities, such as irrigation and playing on contaminated soil, compared with their female counterparts.

### 4.1. Limitations of the Study

Although this study revealed important findings about STH among schoolchildren, it had some limitations. First, a single stool sample was obtained from each study participant and processed with a single Kato–Katz thick fecal smear for microscopy. This might underestimate the actual prevalence of STHs among the studied schoolchildren in the study area. Second, most of the schools are located in rural areas which lack electricity for reading of Kato–Katz plates within sixty minutes. This situation might underestimate the true prevalence of STH particularly for hookworm infection.

## 5. Conclusion

This study revealed that nearly 40% of schoolchildren in the study area were positive for one or more STH. Hookworm was the most prevalent STH followed by *A. lumbricodes* and *T. trichiura.* Among the risk factors considered in the present study, male gender, lack of toilet, playing on soil, having untrimmed fingernails, engagement in irrigation, walking barefoot, and lack of knowledge on the prevention and control methods were found to be independent explanatory factors for STH infection among schoolchildren around Lake Tana. This highlights the importance of integrated STH control strategies including improved awareness, construction of toilets, and hand washing facilities. WASH program should be expanded to all endemics areas together with the ongoing MDA program.

## 6. Declarations

We declared that this article is our original work and this manuscript is not submitted to other journals elsewhere.

## Figures and Tables

**Table 1:** Classification of soil-transmitted helminth (STH) infection intensity based on World Health Organization guidelines.

Types of STHs	Class of infection intensity based on eggs per gram		
	Light	Moderate	Heavy
*Ascaris lumbricoides*	1–4,999	5,000–49,999	>49,999
*Trichuris trichiura*	1–999	1,000–9,999	>9,999
Hookworms	1–1,999	2,000–3,999	>3,999

**Table 2:** Prevalence of soil-transmitted helminth infection across sociodemographic characteristics of the schoolchildren around Lake Tana, northwest Ethiopia, 2021.

Variables	Infection status of school children		
	Positive (%)	Negative (%)	Total (%)

Gender			
Female	57 (32.0)	121 (68.0)	178 (52.8)
Male	72 (45.3)	87 (54.7)	159 (47.2)
Age ranges (years)			
5–9	26 (32.5)	54 (67.5)	80 (23.7)
10–14	87 (39.0)	136 (61.0)	223 (66.2)
15–19	16 (47.1)	18 (52.9)	34 (10.1)
Grade levels			
1–4	66 (32.8)	135 (67.2)	201 (59.6)
5–8	63 (46.3)	73 (53.7)	136 (40.4)
Residence			
Urban	7 (10.9)	57 (89.1)	64 (19.0)
Rural	122 (44.7)	151 (55.3)	273 (81.0)
Family size			
1–3	19 (40.4)	28 (59.6)	47 (13.9)
4–6	48 (28.6)	120 (71.4)	168 (49.9)
Above 6	62 (50.8)	60 (49.2)	122 (36.2)
Religion			
Orthodox	128 (38.8)	202 (61.2)	330 (97.9)
Muslim	1 (14.3)	6 (85.7)	7 (2.1)
Mother's education			
Illiterate	79 (43.4)	103 (56.6)	182 (54.0)
Primary school	42 (37.5)	70 (62.5)	112 (33.2)
Secondary school	7 (17.9)	32 (82.1)	39 (11.6)
College and above	1 (25.0)	3 (75.0)	4 (1.2)
Father's education			
Illiterate	115 (47.5)	127 (52.5)	242 (71.8)
Primary school	12 (18.5)	53 (81.5)	65 (19.3)
Secondary school	1 (3.8)	25 (7.4)	26 (4.4)
College and above	1 (25.0)	3 (75.0)	4 (1.2)
Family occupation			
Farmer	101 (43.5)	131 (56.5)	232 (68.8)
Merchant	23 (29.1)	56 (70.9)	79 (23.4)
Employed	2 (11.8)	15 (88.2)	17 (5.0)
Others	3 (33.3)	6 (66.7)	9 (2.7)
Residence			
Rural	122 (94.6)	151 (72.6)	273 (81.0)
Urban	7 (5.4)	57 (27.4)	64 (19.0)
Schools			
Robit	48 (45.7)	57 (54.3)	105 (31.1)
St. Hana	33 (44.0)	42 (56.0)	75 (22.3)
Woramit	27 (38.6)	43 (61.4)	70 (20.8)
Kunzila	15 (22.4)	52 (77.6)	67 (19.9)
Gurer	6 (30.0)	14 (70.0)	20 (5.9)
			
Total	129 (38.3)	208 (61.7)	337 (100%)
			

**Table 3:** Prevalence of soil-transmitted helminth (STH) infection among the selected primary schools in and around Lake Tana, 2021.

Name of the schools	Hookworm, No. (%)	*Ascaris lumbricoides*, No. (%)	*Trichuris trichiura*, No. (%)	All STHs, No. (%)

Robit	45 (12.8)	4 (1.2)	0 (0)	48 (14.2)
St. Hana	21 (6.2)	19 (5.6)	2 (0.6)	33 (9.8)
Woramit	14 (4.2)	13 (3.9)	2 (0.6)	27 (8.0)
Kunzila	4 (1.2)	11 (3.3)	0 (0)	15 (4.5)
Gurer	4 (1.2)	1 (0.3)	1 (0.3)	6 (1.8)
				
Total	88 (26.1)	48 (14.2)	5 (1.5)	129 (38.3)
				

**Table 4:** Univariate and multivariate logistic regression analyses for sociodemographics and other potential risk factors associated with soil-transmitted helminths (STHs) infections around Lake Tana, 2021.

Variables	STHs		Crude OR	*p*-value	Adjusted OR	*p*-value
	Positives, No. (%)	Negative, No. (%)				
Gender						
Female	57 (44.2)	121 (58.2)	1		1	
Male	72 (55.8)	87 (41.8)	1.757 (1.128–2.737)	0.013	2.286 (1.191–4.388)	0.013
Age ranges (years)						
5–9	26 (32.5)	54 (67.5)	1			
10–14	87 (39.0)	136 (61.0)	1.329 (0.774–2.279)	0.302		
15–19	16 (47.1)	18 (52.9)	1.955 (0.854–4.472)	0.112		
Grade						
1–4	66 (32.8)	135 (67.2)	1		1	
5–8	63 (46.3)	73 (53.7)	1.765 (1.128–2.762)	0.013	2.615 (1.260–5.428)	0.010
Residence						
Urban	7 (10.9)	57 (89.1)	1		1	
Rural	122 (44.7)	151 (55.3)	6.579 (2.896–14.944)	0.000	1.981 (0.605–6.484)	0.259
Family size						
1–3	19 (40.4)	28 (59.6)	1		1	
4–6	48 (28.6)	120 (71.4)	0.589 (0.301–1.154)	0.123	0.335 (0.125–0.897)	0.030
Above 6	19 (40.4)	28 (59.6)	1.523 (0.770–3.012)	0.227	1.110 (0.422–2.918)	0.832
Family occupation						
Employed	2 (11.8)	15 (88.2)	1		1	
Others	3 (33.3)	6 (66.7)	3.750 (0.495–28.389)	0.201	7.265 (0.385–137.263)	0.186
Merchant	23 (29.1)	56 (70.9)	3.080 (0.652–14.560)	0.156	3.284 (0.302–35.755)	0.329
Farmer	101 (43.5)	131 (56.5)	5.782 (1.293–5.865)	0.022	8.731 (0.8.3–94.953)	0.075
Mother's education						
College and above	1 (25.0)	3 (75.0)	1			
Secondary	1 (3.8)	25 (96.2)	0.120 (0.006–2.458)	0.169		
Primary	12 (18.5)	53 (81.5)	0.679 (0.065–7.110)	0.747		
Illiterate	115 (47.5)	127 (52.5)	2.717 (0.279–26.484)	0.390		
Father's education						
College and above	1 (25.0)	3 (75.0)	1			
Secondary	7 (17.9)	32 (82.1)	0.656 (0.059–7.280)	0.732		
Primary	42 (37.5)	70 (62.5)	1.800 (0.181–17.868	0.616		
Illiterate	79 (43.4)	103 (56.6)	2.301 (0.235–22.543)	0.474		
Hand washing facility in school						
Yes	57 (31.8)	122 (68.2)	1		1	
No	72 (45.6)	86 (54.4)	1.792 (1.150–2.793)	0.010	1.483 (0.664–3.313)	0.337
Knowledge on the route of STH transmission						
Yes	61 (32.6)	126 (67.4)	1		1	
No	68 (45.3)	82 (54.7)	1.713 (1.099–2.669)	0.017	1.696 (0.779–3.693)	0.184
Habit of eating un-washing vegetables						
No	81 (34.5)	154 (65.5)	1		1	
Yes	48 (47.1)	54 (52.9)	1.690 (1.053–2.712)	0.030	1.981 (0.986–3.982)	0.055
Knowledge on prevention and control of STHs						
Yes	24 (24.0)	76 (76.0)	1		1	
No	105 (44.3)	132 (55.7)	2.519 (1.489–4.261)	0.001	5.407 (2.440–11.979)	<0.001
Irrigation						
No	79 (33.3)	158 (66.7)	1		1	
Yes	50 (50.0)	50 (50.0)	2.000 (1.242–3.219)	0.004	2.140 (1.002–4.569)	0.049
Source of water						
Tap water	44 (28.8)	109 (71.2)	1			
Spring	58 (42.0)	80 (58.0)	3.303 (1.552–7.031)	0.002	1.458 (0.468–4.544)	0.516
River	7 (63.6)	4 (36.4)	4.335 (1.209–15.551)	0.024	2.080 (0.239–18.087)	0.507
Ground water	20 (57.1)	13 (42.9)	1.796 (1.104–2.922)	0.018	0.610 (0.276–1.347)	0.221
Shoes wearing habit						
Always	8 (10.7)	67 (89.3)	1			
Sometimes	66 (38.4)	106 (61.6)	5.215 (2.355–11.547)	0.000	6.855 (1.625–28.914)	0.009
Not at all	55 (61.1)	35 (38.9)	13.161 (5.643–30.694)	0.000	29.576 (6.599–132.548)	<0.001
Availability of toilet						
Yes	58 (27.8)	151 (72.2)	1		1	
No	71 (55.5)	57 (44.5)	3.243 (2.044–5.146)	0.000	3.059 (1.306–7.162)	0.010
Defecation in an open field						
No	35 (24.5)	108 (75.5)	1		1	
Yes	94 (48.5)	100 (51.5)	2.901 (1.806–4.659)	0.000	0.952 (0.398–2.280)	0.912
Washing hands after toilet						
Always	16 (21.6)	58 (78.4)	1		1	
Sometimes	30 (41.1)	39 (53.4)	2.580 (1.382–4.816)	0.003	0.530 (0.125–2.252)	0.390
Not at all	83 (43.6)	111 (58.4)	3.160 (1.539–6.490)	0.002	0.210 (0.045–0.971)	0.046
Playing with soil						
No	35 (21.0)	132 (79.0)	1			
Yes	94 (55.3)	76 (44.7)	4.665 (2.887–7.537)	0.000	5.904 (2.791–12.492)	<0.001
Fingernail trimming						
Yes	47 (33.8)	92 (66.2)	1		1	
No	82 (41.4)	116 (58.6)	1.384 (0.881–2.172)	0.158	3.209 (1.573–6.547)	0.001

AOR: adjusted odds ratio; COR: crude odds ratio; CI, confidence interval.

## Data Availability

Data supporting this research article are available from the corresponding author or first author on reasonable request.

## Abbreviations

SPSS: Statistical Package for Social Science
NTD: Neglected tropical diseases
EPG: Eggs per gram
PGRCSVD: Post Graduate and Research Vice-Dean
WHO: World Health Organization
AOR: Adjusted odds ratio
COR: Crude odds ratio
CI: Confidence interval
MDA: Mass drug administration
RPM: Revolution per minute
SAC: School-age children.
